# Elevated Urinary Titin in Adult Spinal Muscular Atrophy: A Multicenter, Cross-Sectional Observational Study

**DOI:** 10.3390/neurolint17080114

**Published:** 2025-07-22

**Authors:** Andrea Sipos, Emese Rebeka Ripszám, Judit Mária Molnár, Zoltán Grosz, Judit Boczán, Melinda Borbála Altorjay, Livia Dézsi, Anett Csáti, Kristóf Babarczy, Norbert Kovács, Nándor Hajdú, Endre Pál

**Affiliations:** 1 Department of Neurology, Medical School, University of Pécs, 7623 Pécs, Hungary; sipos.andrea@aok.pte.hu (A.S.); kovacs.norbert@pte.hu (N.K.); 2 Institute of Genomic Medicine and Rare Disorders, Semmelweis University, 1085 Budapest, Hungary; ripszam.emese@semmelweis.hu (E.R.R.); molnar.mariajudit@semmelweis.hu (J.M.M.); grosz.zoltan@semmelweis.hu (Z.G.); 3 Department of Neurology, Faculty of Medicine, University of Debrecen, 4032 Debrecen, Hungary; boczan@med.unideb.hu (J.B.); altorjay.melinda@med.unideb.hu (M.B.A.); 4 Department of Neurology, Albert Szent-Györgyi Health Centre, University of Szeged, 6725 Szeged, Hungary; dezsilivia@gmail.com (L.D.); csati.anett@med.u-szeged.hu (A.C.); babarczy.kristof@med.u-szeged.hu (K.B.); 5 Institute of Psychology, Eötvös Loránd University, 1064 Budapest, Hungary; hajdu.nandor93@gmail.com

**Keywords:** biomarkers, spinal muscular atrophy, urinary titin

## Abstract

**Background:** Spinal muscular atrophy (SMA) is a treatable motor neuron disease. Biomarkers for skeletal muscle atrophy are extremely important for measuring the effects of treatment and monitoring the natural course of the disease. The urinary titin N fragment (UNT) has recently been proven to be related to muscle damage. **Methods:** The UNT was measured in 41 patients with SMA and 41 healthy controls. Clinical data, functional tests, and laboratory findings were also recorded. **Results:** We found significantly higher UNT levels in the patient samples than in the healthy subjects. The UNT was not related to disease type, functional test results, or serum creatine kinase levels. **Conclusions:** This cross-sectional study highlights the importance of the UNT as a potential noninvasive biomarker for spinal muscular atrophy. Its role can potentially be verified through longitudinal studies.

## 1. Introduction

Spinal muscular atrophy (SMA) is a motor neuron disorder. It is characterized by progressive muscular atrophy due to loss of motor neurons in the ventral horn of the spinal cord. The vast majority of SMA cases are caused by homozygous deletion of the survival motor neuron 1 (*SMN1*) gene [1]. Globally, SMA occurs in 1 in 10,000 live births, and the prevalence of SMA is 1–2/100,000 individuals, with no differences between sexes [2,3,4].

SMA is classified into five subtypes, from 0 to 4, based on onset and clinical signs. Clinical severity is primarily determined by the copy number of the *SMN2* gene [5]. Possible biomarkers of motoneuron degeneration in SMA include imaging findings (MRI of the spinal cord and muscles), electrophysiologic factors (e.g., motor unit estimation), and circulating proteins, such as SMN protein and neurofilaments [6]. Plasma-phosphorylated neurofilament heavy (pNfH) and light (pNfL) chains are intermediate filaments of neurons that are stable and resistant to degradation, making them ideal for assessing the integrity of neurons and axons. Elevated levels of pNfH and pNfL in the CSF and serum are recognized markers of neuronal degeneration and axon loss [7]. Plasma phosphorylated neurofilament heavy (p-NfH) and light chains (p-NfL) were investigated in SMA, and a rapid decline was found after splicing-modifier therapy was initiated [8]. These biomarkers represent the condition of motor neurons but not skeletal muscles.

Creatine kinase (CK) and serum creatinine are metabolites related to skeletal muscle that have historically been used to estimate myopathy/dystrophy activity and skeletal muscle loss, respectively. These laboratory alterations have been studied in SMA, revealing mildly elevated CK levels and decreased serum creatinine levels in most cases, suggesting moderate ongoing skeletal muscle damage [9]. The release of various sarcomeric, sarcoplasmic, and mitochondrial proteins, including troponins, myosin light chain, myosin binding protein, MYBPC1, titin, fatty acid-binding protein, and myoglobin from muscle fibers into the blood has been investigated [10]. Except for troponin C and myoglobin, these tests are not commercially available. Moreover, biomarkers for assessing disease progression, prognosis, and early response to therapy are unavailable.

Titin is a giant protein expressed in mammals. Different isoforms are found in sarcomeres of the heart and skeletal muscle. Its role is to stabilize and maintain the elasticity of sarcomeres [11].

Urinary titin fragments (UNTs) were discovered in 2014 by Rouillon et al. through proteomic analysis, which showed significantly higher levels in muscular dystrophy patients than healthy controls [12]. In 2016, Maruyama et al. established a sensitive ELISA method [13]. Since then, UNTs have been investigated in several neuromuscular disorders [14].

The purpose of the current study was to investigate UNT levels in adult patients with SMA.

## 2. Materials and Methods

This study was approved by the Medical Research Council (ETT-TUKEB), Hungary. Data on SMA patients were collected from four tertiary neuromuscular centers (Nos. 1 to 4 in the affiliations) participating in the treatment and follow-up of SMA patients in Hungary.

Patients were included if their diagnosis was confirmed through genetic testing, showing a homozygous deletion of exon 7 in the *SMN1* gene. Participants younger than 18 years were excluded. Informed consent was obtained from each participant prior to the start of the study. Urine samples were collected from SMA patients during clinical control visits and before regular treatment and were stored in a freezer at −80 °C until measurement. Control individuals were included based on voluntary applications and had no significant health problems, nor were they taking any drugs. Extreme physical activity was avoided in the week before sample collection. In addition to the common tests, serum CK and urinary creatinine (Cr) levels were measured. The urinary N-terminal fragment of titin (UNT) was determined using an ELISA kit, according to the manufacturer’s instructions (Immuno-Biological Laboratories, Naka Aza-Higashida, Japan, Cat. No. 27900). Titin concentration was normalized to the urinary concentration of creatinine, resulting in a pmol/mg Cr ratio.

Clinical data such as body mass index (BMI), disease duration, number of treatments, and results of functional tests were recorded. These tests include the Six-minute Walk Test (6MWT), Hammersmith Functional Motor Scale Extended (HFMSE), and the Revised Upper Limb Module (RULM). SMA subtypes were defined according to the International SMA consortium meeting [15].

### Statistics

Since the Titin/Cr ratio was not normally distributed, the Mann–Whitney U test was performed. To measure the effect size, Vargha and Delaney’s *A* values were calculated [16]. Kendall’s τ correlation coefficients were calculated to assess correlations between urine Titin/Cr ratio and clinical parameters, such as age, age of onset, BMI, RULM, HFMSE, and 6MWT, which were recorded during regular clinical control at the time of blood and urine collection (JASP software, https://jasp-stats.org/, accessed on 26 November 2024).

## 3. Results

### Patients

Sixty-one patients agreed to participate in this study. However, one patient did not provide a urine sample, and the clinical data for 19 additional patients were not complete during the analysis. Consequently, data and samples from 41 patients and 41 healthy controls were analyzed with age- and sex-matched groups. The basic characteristics of the study participants are presented in Table 1.

**Figure 1:**
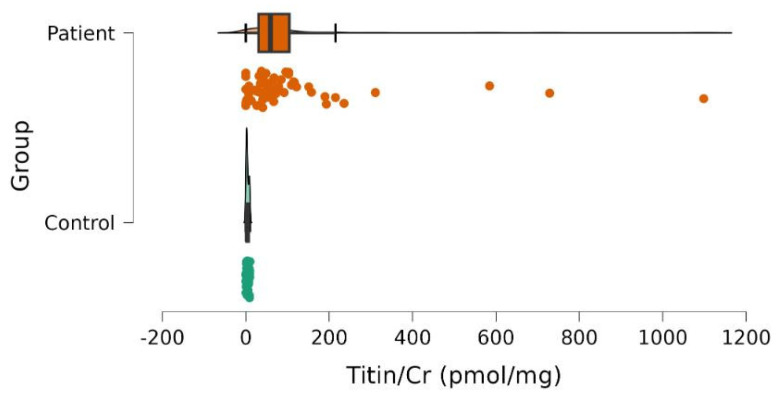
Urinary titin/creatinine (Titin/Cr) values in patientswith SMAand controls. The scatter plot demonstrates the differences between patients (red) and controls (green). Color dots correspond to individual subjects. Significantly higher values were observed in patients.

We observed significantly higher UNT levels among patients with SMA compared to healthy controls: median ± MAD (min–max) was 52.2 ± 65.0 (0.0–1098.5) and 3.2 ± 3.1 (0.0–9.9) pmol/mgCr, respectively, *p* < 0.001 (Figure 1).

To evaluate the effect of treatment, we compared the UNT and clinical data between treated (*n* = 30) and untreated (*n* = 10) SMA patients. No significant differences were found in clinical data (e.g., duration, serum CK, HFMSE, RULM, and 6MWT) or UNT levels, although UNT levels were lower in samples from treated subjects (median ± SE (min–max) in untreated/treated patients were 82.3 ± 0.7 (0.0–310.8) and 54.0 ± 0.3 (0.0–1098.5), respectively).

**Figure 2:**
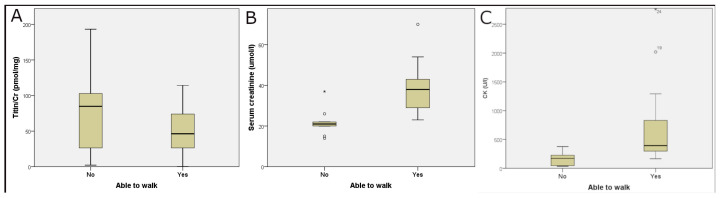
Effect of walking ability on laboratory parameters. UTN (Titin/Cr) levels were not statistically different (**A**), but serum creatinine (**B**) and creatine kinase (CK, (**C**)) levels were significantly higher in patients who had preserved walking ability than in wheelchair-bound patients. Circles outside the boxes represent data points that fall more than 1.5 × interquartile range.

There was no significant difference in the UNT levels among patients belonging to SMA type 2 and type 3. We examined the significance of walking ability in relation to biochemical parameters. In patients with preserved walking ability, UNT levels were lower (non-significant), but serum creatinine and CK levels were significantly higher than in wheelchair-bound patients (Table 2 and Figure 2).

**Figure 3:**
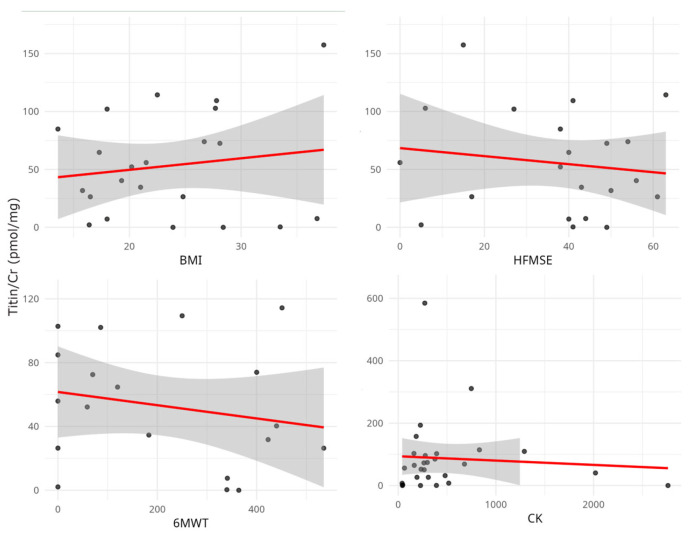
Relationship between urinary titin level (Titin/Cr) and clinical and laboratory parameters. Scatterplots represent individual patient data. No significant correlations were observed between urinary titin/Cr value, functional tests, and CK. CK, creatinine kinase; BMI, body mass index; HFMSE: Hammersmith Functional Motor Scale Extended; 6MWT, Six-minute Walk Test.

Correlation analysis revealed a marginally positive correlation between UNT levels and CK (r = 0.25, *p* = 0.06) but not between UNT levels and other clinical data (Figure 3). Serum creatinine (sCr) and CK levels were strongly correlated with the functional tests (Table 3).

The table shows the correlations (r) among clinical parameters, creatine kinase (CK), and serum creatinine (sCr) levels. All values were statistically significant (*p* < 0.001).

## 4. Discussion

SMA has recently become a treatable motor neuron disease, and the clinical course of the disease has changed significantly with the introduction of new treatment options. This development highlights the need for more precise monitoring of patients’ motor skills and quality of life. The HFMSE, RULM, and 6MWT are widely used and validated scores for assessing motor function in patients with SMA. However, they have limitations in everyday practice: The RULM exhibits a “ceiling” effect in mildly impaired patients, while severely disabled patients are unable to perform tasks, resulting in a “flooring” effect in the HFMSE. In addition, clinical changes can be difficult to measure in certain cases. Gait and 6MWT are influenced by factors beyond the musculature, such as lung capacity and skeletal deformities [17,18]. Therefore, multiple clinical and biochemical markers are recommended for the reliable assessment of disease course and progression. Previous biomarker studies have focused on motor neurons and axonal damage, which is the main pathology of SMA. Neurofilament level appears to be a clinically useful marker of motor neuron health and degeneration, making it suitable for therapy follow-up, although its measurement is expensive and not widely available [7,8]. With regard to skeletal muscle degeneration, only a limited number of markers are available in clinical practice. Generally, serum CK is a marker of disease activity, and serum creatinine levels reflect muscle mass, neither of which indicates disease severity. In SMA, a recent study found that the serum CK level was elevated in 33.1% of patients, while the creatinine level was decreased by 90.1%. Clinical severity data (HSFME and RULM but not 6MWT) were correlated with serum CK and creatinine levels. Interestingly, SMA type 3 and ambulatory patients had higher CK levels [8], which might indicate higher disease activity, whereas in advanced stages, CK is not informative. Our results showed similarly strong correlations between functional tests, serum CK, and sCr but not with the UNT. Our study confirmed that walkers had higher CK and serum creatinine levels. Elevated CK levels suggest high disease activity in the early stages of the disease. Furthermore, low creatinine levels may correspond to a more pronounced loss of skeletal muscle in the advanced wheelchair-bound stage.

Recent studies have suggested that urinary titin/creatinine ratio is a valuable biomarker of skeletal and heart muscle damage. Our previous study showed that UNT levels were significantly elevated in patients with myotonic dystrophy type 1 compared to healthy individuals [19]. In previous reports, UNT levels were determined in various conditions. In a large cohort of patients with Duchenne muscular dystrophy, values were nearly 700 times higher, and in Becker muscular dystrophy, they were 100 times higher than those in controls. UNT levels decreased with age and were correlated with serum CK levels. Ambulatory patients exhibited higher UNT levels than wheelchair-bound patients; however, steroid therapy did not significantly affect UNT levels [20,21].

Acute exercise led to a 2-fold increase after concentric exercise but a 45-fold increase after eccentric exercise, indicating that eccentric muscle injury is more severe [22]. Significantly high UNT levels have been observed in autoimmune myositis, typically associated with severe skeletal muscle injury [23]. The UNT has been reported as a predictor of cardiac and all-cause mortality in dilated cardiomyopathy [24]. Interestingly, sarcopenia causes a moderate increase in UNT levels [25,26]. Regarding motor neuron diseases, in our study, we found a more than 15-fold increase in UNT levels in SMA patients compared with controls. The UNT was recently investigated in amyotrophic lateral sclerosis (ALS), where a five-fold higher value was measured than in healthy controls [27]. Although ALS and SMA differ in their pathomechanisms, denervation plays a common role. Our results align with those of previous ALS studies. In the absence of innervation, the skeletal muscle undergoes progressive atrophy, ultimately resulting in the replacement of functional muscle tissue with fibrous connective tissue and fat. This process spans months to years in humans. Denervation initiates a cascade of events that lead to muscle atrophy and structural alterations. Following immediate loss of function, this process later disrupts the integrity of sarcomeres, the fundamental functional units of muscle fibers. As sarcomeres deteriorate, numerous structural proteins, including titin, which is essential for maintaining sarcomere structure and elasticity, are degraded. In animal models, a more significant loss of titin compared to myosin heavy chain (MHC) and actin content has been observed in atrophic muscles. The ultrastructure of myofibrils also reveals disturbed arrangements of myofilaments and a disorganized contractile apparatus in denervated muscles. Titin degradation proceeds via various mechanisms, including ubiquitination, calcium-dependent proteolysis, phosphorylation, and autophagy [28,29,30]. The degraded protein fragments are released into the bloodstream. Subsequently, titin fragments are filtered by the kidneys and excreted in urine, serving as a biomarker for muscle damage. The inclusion of neurofilament data in an extended biomarker panel could provide additional insights into the extent of neuronal damage accompanying muscle denervation, thereby offering a more comprehensive assessment of neuromuscular health.

In summary, according to previous studies and our observations, the UNT is a marker of both skeletal muscle tissue damage and loss. Sarcomeric injury is the primary process in acute/subacute skeletal muscle diseases, leading to the release of CK from muscle, its elevation in serum, and excretion in urine as the UNT, with these factors being interrelated. Conversely, sarcomeric injury and muscle atrophy progress concurrently in chronic conditions, but the correlation between the UNT and CK release is weak.

The limitations of our study include the fact that most of our patients received splicing-modifier therapy, with only a small proportion untreated. Although we found lower UNT levels in treated patient samples, it was not significant, and a low element number might have influenced this result. This is a cross-sectional study. Serial measurements of UNT during 1 to 3 years of follow-up may provide valuable insights into disease progression.

## 5. Conclusions

Our results may offer a new, easy-to-use fluid biomarker for SMA to monitor muscle damage. Comparing the dynamics of motor neuron loss and skeletal muscle atrophy might provide further insights into the disease’s pathomechanism. This study completes previous investigations of skeletal muscle damage, which might develop under different circumstances, either due to direct effects (injury), degeneration (muscular dystrophy), or in a secondary way, such as denervation, as seen in SMA.

## Figures and Tables

**Table 1:** Basic characteristics of subjects.

	Patients (*n* = 41)	Controls (*n* = 41)
Age * (years)	34.1 (18–64)	39.7 (18–57)
Sex (female/male)	17/24	18/23
BMI **	23.3 ± 6.3	22.7 ± 6
SMA type		
2	8	NA
3	31	NA
4	2	NA
HFMSE **	35.0 ± 18.4	NA
RULM **	29.6 ± 8.8	NA
6MWT ** (meters)	290.1 ± 160.3	NA
Treated ***	31	NA

*: mean (minimum–maximum), **: mean ± SD, ***: number of patients treated with splicing modifiers, BMI: body mass index, HFMSE: Hammersmith Functional Motor Scale Extended, NA: Not Applicable, RULM: Revised Upper Limb Module, 6MWT: Six-minute Walk Test.

**Table 2:** Effect of walking ability on clinical scores and biochemical parameters.

Walking Ability	No	Yes	Significance
**Age (Years)**	35.9 ± 13.4	36.2 ± 14.1	n.s.
**Duration (Months)**	29.5 ± 13.8	26.7 ± 12.7	n.s.
**BMI (kg/m^2^)**	22.8 ± 7.5	23.7 ± 6.6	n.s.
**HFMSE**	16.4 ± 14.9	**46.9 ± 9.7**	***p* < 0.001**
**Titin/Cr (pmol/mg)**	80.7 ± 65.6	52.1 ± 38.8	n.s.
**Serum Creatinine (umol/L)**	21.8 ± 6.7	**38.5 ± 13.2**	***p* < 0.001**
**Serum CK (U/L)**	160.3 ± 114.7	**765.4 ± 794.8**	***p* < 0.001**

As anticipated, the patients who retained their ability to walk exhibited significantly higher HFMSE scores than those confined to wheelchairs. Although the urinary Titin/Cr concentration was elevated in the non-ambulant group, the difference was not statistically significant (n.s.). Despite having comparable BMI, ambulant patients demonstrated significantly higher levels of serum creatinine and creatine kinase (CK).

**Table 3:** Correlations between motor functions and biochemical parameters.

	CK	sCr
**HFMSE**	0.707	0.781
**RULM**	0.775	0.718
**6MWT**	0.734	0.840

## Data Availability

Raw data supporting the conclusions of this study will be made available by the authors upon request.
